# Infant Botulism

**DOI:** 10.21980/J88350

**Published:** 2023-07-31

**Authors:** Ashley Garispe, Steven Cherry

**Affiliations:** * Vituity Healthcare and Medical Staffing Services, Saint Agnes Medical Center, Department of Emergency Medicine, Fresno, CA; ^ Vituity Healthcare and Medical Staffing Services, Sutter Roseville Medical Center, Department of Emergency Medicine, Roseville, CA

## Abstract

**Audience:**

This oral board case is appropriate for emergency medicine residents and medical students (with senior resident assistance) on emergency medicine rotation.

**Introduction:**

Although a somewhat rare disease, infant botulism is a true pediatric emergency that carried a 90% rate of mortality prior to the development of an antitoxin.^1^ While botulism infections can be iatrogenic, foodborne, or involve infected wounds, infant botulism remains the most common presentation of this disease and accounts for approximately 70% of new cases annually.^2^ Caused by *Clostridium botulinum*, the inactive spores are ingested by the infant and germinate in the large intestine.^3^,^4^ The resulting neurotoxin prevents the release of acetylcholine at the presynaptic membrane which results in flaccid paralysis. Classically, the bulbar musculature is affected before somatic muscular, which results in the typical presentation of “descending paralysis.”^2^,^5^ While confirmatory testing is important, it is often delayed by more than 24 hours, making both clinical recognition and implementation of treatment before confirmatory testing of vital importance.^6^,^7^ Treatment consists of providing airway, nutritional, and hydration support in addition to administering botulinum-specific antitoxin.^8^,^9^ While patients over the age of 12 months are treated with equine botulinum antitoxin, the Food and Drug Administration (FDA) has approved a human-derived immunoglobulin treatment, Botulism Immune Globulin Intravenous (BIG-IV, ie, “Baby BIG”) for pediatric patients less than 12 months of age.^1^,^2^,^6^ Ordering BIG-IV is a complex and multidisciplinary process, requiring the treating physician to discuss any suspicious case with the Infant Botulism Treatment and Prevention Program (IBTPP) which is a branch of the California Department of Public Health.^6^ With early recognition and implementation of treatment, most infants will make a full recovery.

**Educational Objectives:**

At the end of this oral board session, examinees will: 1) demonstrate an ability to obtain a complete pediatric medical history, 2) perform an appropriate physical exam on a pediatric patient, 3) investigate a broad differential diagnosis for neuromuscular weakness in a pediatric patient, 4) recognize the classic presentation of infant botulism and implement treatment with botulinum specific antitoxin before confirmatory testing, 5) recognize impending airway failure and intubate the pediatric patient with appropriately dosed medications and ET tube size, and 6) demonstrate effective communication with healthcare team members and parents.

**Educational Methods:**

This oral board case followed the standard American Board of Emergency Medicine-style case in a tertiary care hospital with access to all specialists and resources needed. This case was tested using 12 resident volunteers ranging from PGY 1–2 in an ACGME (Accreditation Council for Graduate Medical Education) accredited emergency medicine residency program. Learners were debriefed immediately after the case and were given the opportunity to provide feedback.

**Research Methods:**

The learners participating in the oral board case provided immediate feedback both by verbal discussion and via a written survey requiring them to rate the efficacy of the exercise. The efficacy of the educational content was assessed by comparing scoring measures of the ACGME core competencies across all learners based on post graduate year (PGY). Scoring measures were determined using a scale from 1–8, with 1–4 being unacceptable performance and 5–8 being acceptable. Efficacy required full completion of the oral board case by the residents as well as a debriefing session during which key educational concepts were discussed.

**Results:**

The practice oral board candidates consisted of 7 PGY1 and 5 PGY2 level residents. The average score of participating residents for each training level was PGY1: 4.5 and PGY2: 5.7. All except for 2 PGY2 residents missed at least one critical action with the majority of PGY1 residents missing more than one critical action for the case. All participating residents rated the educational value of the case as 4.75 (1–5 Likert scale, with 5 being excellent).

**Discussion:**

The educational content of this oral board case and debriefing session were effective for teaching the presentation, evaluation, and appropriate management of infant botulism. Infant botulism is a true pediatric emergency and prompt recognition and treatment is imperative in order to decrease mortality. While mortality was approximately 90% one hundred years ago, today infant botulism carries a much better prognosis due to the advent of antitoxin treatment with a mortality closer to 15%.^1^ This case highlights several classic physical exam findings including bulbar findings in addition to somatic weakness. Additionally, this case requires definitive airway management with endotracheal intubation, which is true for approximately 50% of infants with botulism.^1^ While a stool culture or direct toxin assay of the gastric contents, serum, or stool should be performed to confirm the diagnosis, these tests are often performed by the state health department or the Centers for Disease Control (CDC) and often take up to five days to result, during which time the patient will continue to deteriorate. Therefore, the treating physician should seek emergent consultation with the IBTPP to help facilitate the multidisciplinary decision to initiate treatment with human-derived anti-botulinum toxin antibodies.^6^ If the IBTPP deems that infant botulism is highly suspected based on the history and physical exam, then appropriate treatment should not be delayed and BIG-IV should be administered.^6^, ^7^ With early recognition and implementation of treatment, most infants will make a full recovery within several months to a year. Upon discharge, patients will likely require outpatient neurology follow-up in addition to physical therapy to aid in recovery. Because infant botulism is a true pediatric emergency with potentially high mortality, reaching the appropriate diagnosis expeditiously will allow the emergency physician to communicate effectively with worried parents regarding the disease progression and facilitate correct treatment early in order to prevent significant sequela.

**Topics:**

Pediatric weakness, pediatric neurotoxin, infant botulism, neuromuscular weakness.

## USER GUIDE

[Table t1-jetem-8-3-o32]

**Table t1-jetem-8-3-o32:** 

List of Resources:	
Abstract	33
User Guide	36
For Examiner Only	38
Oral Boards Assessment	45
Stimulus	48
Debriefing and Evaluation Pearls	60

**Learner Audience:**

Medical students, interns, junior residents, senior residents

**Time Required for Implementation:**

Case: 15 minutes

Debriefing: 10 minutes

**Learners per instructor:**

One resident per instructor (or one medical student and resident combination per instructor)

**Topics:**

Pediatric weakness, pediatric neurotoxin, infant botulism, neuromuscular weakness.

**Objectives:**

At the end of this oral board session, examinees will:

1. Demonstrate an ability to obtain a complete pediatric medical history
2. Perform an appropriate physical exam on a pediatric patient
3. Investigate a broad differential diagnosis for neuromuscular weakness in a pediatric patient
4. Recognize the classic presentation of infant botulism and implement treatment with botulinum specific antitoxin before confirmatory testing
5. Recognize impending airway failure and intubate the pediatric patient with appropriately dosed medications and ET tube size
6. Demonstrate effective communication with healthcare team members and parents.

### Linked objectives and methods

The oral boards simulation method was selected to aid the learners in accomplishing the above learning objectives as it provides an opportunity to experience and manage a relatively rare clinical scenario. Through this format, learners are challenged to verbalize how they would obtain a complete pediatric history and physical exam (objectives 1 and 2). They need to consider a broad differential to come to the appropriate diagnosis (objective 3). Furthermore, they are challenged to not only recognize a classic presentation of infant botulism (objective 4), but must also manage a common sequela (respiratory failure) and initiate management without a definitive diagnosis by facilitating Human BIG-IV administration in conjunction with the IBTPP before confirmatory tests have resulted (objectives 5 and 6).

### Recommended pre-reading for instructor

The instructor would benefit from reviewing the debriefing slides for this case as well as the Department of Public Health website overviewing the treatment and prevention of infant botulism. There is also a comprehensive summary article entitled “Infantile Botulism” on StatPearls online bookshelf that would be beneficial to review prior to administering the case. Citations for these sources can be founded under “references.”

- Department of Public Health. *Welcome to the Infant Botulism Treatment and Prevention Program*, 2010, https://www.infantbotulism.org/general/babybig.php
- Van Horn NL, Street M. Infantile botulism. [Updated 2022 Jun 21]. In: StatPearls [Internet]. Treasure Island (FL): StatPearls Publishing; 2022 Jan-. Available from: https://www.ncbi.nlm.nih.gov/books/NBK493178/

### Results and tips for successful implementation

This case is best implemented by having one instructor per learner in each session. This case was conducted in a round-robin fashion, with learners rotating through an individual instructor’s station one at a time. Learners had 15 minutes to complete the case followed by a 10-minute debriefing session in which the instructor provided feedback specific to the learner’s performance and also reviewed the debriefing PowerPoint slides. Focusing the debriefing session on resident feedback and missed critical action(s) allowed residents more time to address their individual knowledge gaps.

This case was administered to 7 PGY1 and 5 PGY2 level residents. All residents that participated in the case were able to identify infant botulism as the most likely diagnosis; however, they displayed varying degrees of knowledge regarding both definitive diagnostic testing and treatment. All except for 2 PGY2 residents missed at least one critical action; the majority of PGY1 residents missed more than one critical action. The participating residents rated the educational value of the case as 4.75 (1–5 Likert scale, with 5 being excellent). The efficacy of the educational content was assessed by comparing scoring measures of the ACGME core competencies across all learners based on their post-graduate year. Scoring measures were determined using a scale from 1–8, with 1–4 being unacceptable performance and 5–8 being acceptable. The average score of participating residents for each training level was PGY1: 4.5 and PGY2: 5.7.

## Supplementary Information



## Figures and Tables

**Table t2-jetem-8-3-o32:** 

**Vitals:**	HR 120	BP 96/22	RR 16	Rectal Temp 37.5°C (99.6°F)	O_2_ Sat 93%

**Table t3-jetem-8-3-o32:** 

**Weight:** 7.9 kg (17 lbs 8 oz)	**Height:** 68 cm (26.8 inches)

**Table t4-jetem-8-3-o32:** 

	Milestone	Did not achieve level 1	Level 1	Level 2	Level 3
**1**	**Emergency Stabilization (PC1)**	□ **Did not achieve Level 1**	□ **Recognizes abnormal vital signs**	□ **Recognizes an unstable patient, requiring intervention** **Performs primary assessment** **Discerns data to formulate a diagnostic impression/plan**	□ **Manages and prioritizes critical actions in a critically ill patient** **Reassesses after implementing a stabilizing intervention**
**2**	**Performance of focused history and physical (PC2)**	□ **Did not achieve Level 1**	□ **Performs a reliable, comprehensive history and physical exam**	□ **Performs and communicates a focused history and physical exam based on chief complaint and urgent issues**	□ **Prioritizes essential components of history and physical exam given dynamic circumstances**
**3**	**Diagnostic studies (PC3)**	□ **Did not achieve Level 1**	□ **Determines the necessity of diagnostic studies**	□ **Orders appropriate diagnostic studies** **Performs appropriate bedside diagnostic studies/procedures**	□ **Prioritizes essential testing** **Interprets results of diagnostic studies** **Considers risks, benefits, contraindications, and alternatives to a diagnostic study or procedure**
**4**	**Diagnosis (PC4)**	□ **Did not achieve Level 1**	□ **Considers a list of potential diagnoses**	□ **Considers an appropriate list of potential diagnosis** **May or may not make correct diagnosis**	□ **Makes the appropriate diagnosis** **Considers other potential diagnoses, avoiding premature closure**
**5**	**Pharmacotherapy (PC5)**	□ **Did not achieve Level 1**	□ **Asks patient for drug allergies**	□ **Selects an appropriate medication for therapeutic intervention, considering potential adverse effects**	□ **Selects the most appropriate medication(s) and understands mechanism of action, effect, and potential side effects** **Considers and recognizes drug-drug interactions**
**6**	**Observation and reassessment (PC6)**	□ **Did not achieve Level 1**	□ **Reevaluates patient at least one time during the case**	□ **Reevaluates patient after most therapeutic interventions**	□ **Consistently evaluates the effectiveness of therapies at appropriate intervals**
**7**	**Disposition (PC7)**	□ **Did not achieve Level 1**	□ **Appropriately selects whether to admit or discharge the patient**	□ **Appropriately selects whether to admit or discharge** **Involves the expertise of some of the appropriate specialists**	□ **Educates the patient appropriately about their disposition** **Assigns patient to an appropriate level of care (ICU/Tele/Floor)** **Involves expertise of all appropriate specialists**
**22**	**Patient centered communication (ICS1)**	□ **Did not achieve level 1**	□ **Establishes rapport and demonstrates empathy to patient (and family)** **Listens effectively**	□ **Elicits patient’s reason for seeking health care**	□ **Manages patient expectations in a manner that minimizes potential for stress, conflict, and misunderstanding.**
**23**	**Team management (ICS2)**	□ **Did not achieve level 1**	□ **Recognizes other members of the patient care team during case (nurse, techs)**	□ **Communicates pertinent information to other healthcare colleagues**	□ **Communicates a clear, succinct, and appropriate handoff with specialists and other colleagues** **Communicates effectively with ancillary staff**

**Table t5-jetem-8-3-o32:** 

**#1**	**Patient Information Form**
**#2**	**Monitor Strip**
**#3**	**12 Lead EKG**
**#4**	**Portable Chest x-ray – post intubation**
**#5**	**Portable Chest x-ray – post repositioning of ET tube**
**#6**	**CBC**
**#7**	**Chemistry**
**#8**	**Venous Blood Gas and Lactate**
**#9**	**Urinalysis**
**#10**	**CT Head without Contrast**
**#11**	**Influenza, RSV, COVID-19 Viral Swabs**

**Table t6-jetem-8-3-o32:** 

**Patient’s Name:**	**Lily Pond**	
**Age:**	**6 months old**	
**Gender:**	**Female**	
**Chief Complaint:**	**Lethargy**	
**Person Providing History:**	**Mother**	
**Vital Signs:**		
**Rectal Temp:**	**37.5°C (99.6°F)**	
**BP:**	**96/52**	
**P:**	**120**	
**RR:**	**16**	
**Pulse Ox:**	**93% (room air)**	
**Weight:**	**7.9 kg (17 lbs, 8 oz)**	**Height: 68 cm (26.8 inches)**

**Figure f1-jetem-8-3-o32:**
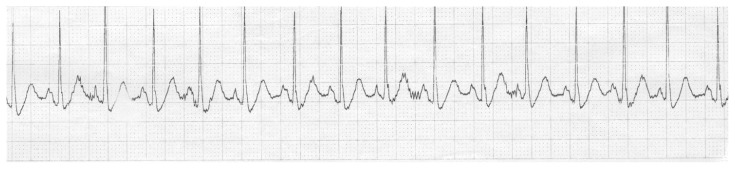


**Figure f2-jetem-8-3-o32:**
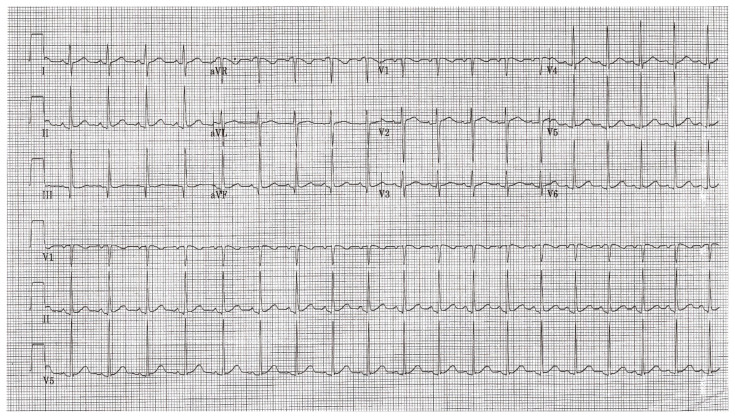


**Figure f3-jetem-8-3-o32:**
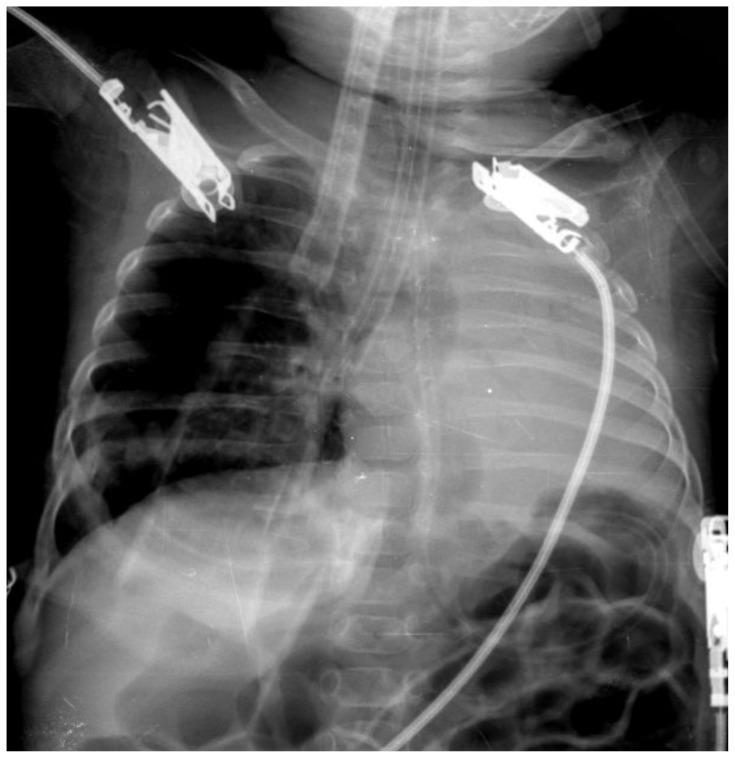


**Figure f4-jetem-8-3-o32:**
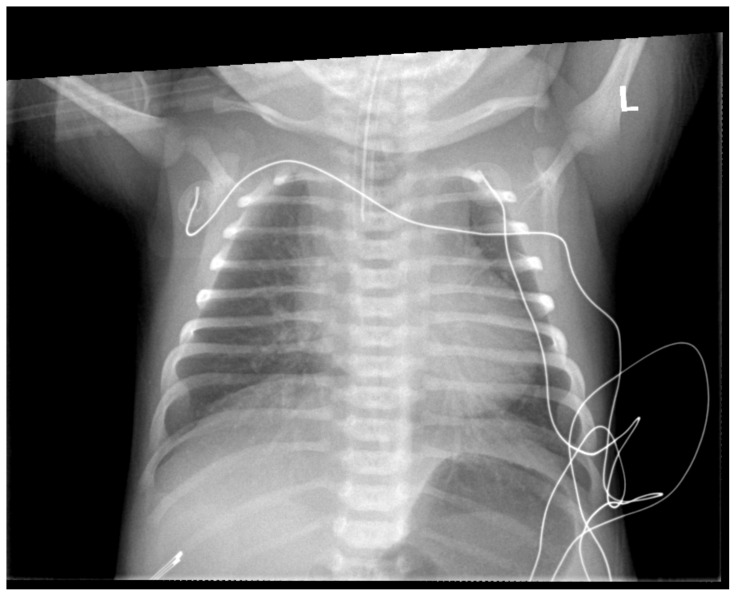


**Table t7-jetem-8-3-o32:** 

**White blood cell count (WBC)**	**14.0 ×1000/mm3**
**Hemoglobin (Hgb)**	**12.0 g/dL**
**Hematocrit (Hct)**	**36.0%**
**Platelets**	**344 ×1000/mm3**
**Segs**	**57%**
**Bands**	**1%**
**Lymphocytes**	**30%**
**Monocytes**	**3%**
**Eosinophils**	**2%**
**Basophils**	**1%**

**Table t8-jetem-8-3-o32:** 

**Sodium**	**140 mEq/L**
**Potassium**	**4.5 mEq/L**
**Chloride**	**106 mEq/L**
**Bicarbonate**	**17 mEq/L (L)**
**Blood Urea Nitrogen (BUN)**	**12 mg/dL**
**Creatinine (Cr)**	**1.2 mg/dL**
**Glucose**	**85 mg/dL**

**Table t9-jetem-8-3-o32:** 

**pH**	**7.30**
**pCO2**	**55 mmHg**
**pO2**	**40 mmHg**
**Bicarb**	**17 mmol/L**
**Lactate**	**2.0 mmol/L**

**Table t10-jetem-8-3-o32:** 

**Color**	**Dark Yellow**
**Appearance**	**Clear**
**Sp Gravity**	**1.105**
**pH**	**6.0**
**Glucose**	**Negative**
**Protein**	**Negative**
**Ketones**	**1+**
**Leukocyte Esterase**	**Negative**
**Nitrite**	**Negative**
**Blood**	**Negative**
**Microscopy**	
**White blood cells (WBC)**	**0–1/hpf**
**Red blood cells (RBC)**	**0–2/hpf**
**Squamous cells**	**0/hpf**
**Bacteria**	**0/hpf**

**Figure f5-jetem-8-3-o32:**
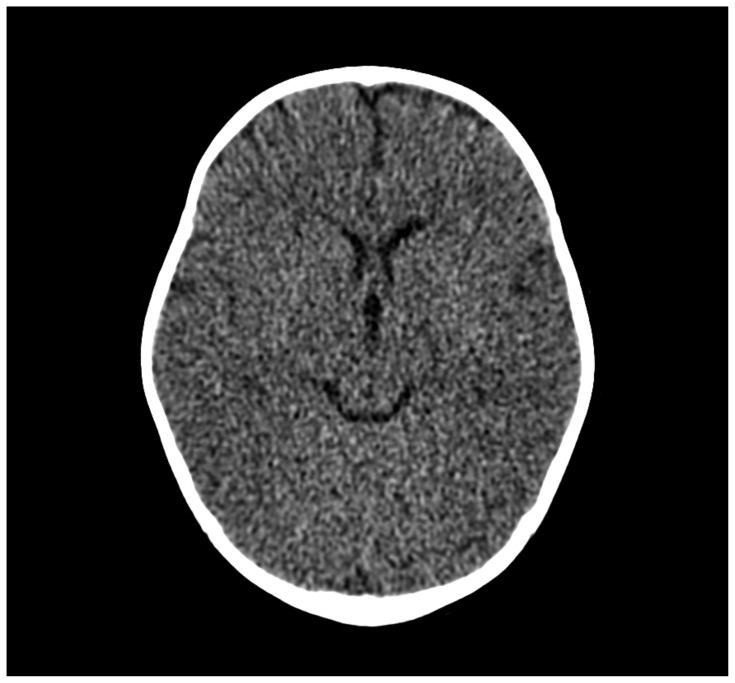


**Table t11-jetem-8-3-o32:** 

**Influenza A**	**Negative**
**Influenza B**	**Negative**
**RSV**	**Negative**
**COVID-19**	**Negative**

**Figure f6-jetem-8-3-o32:**
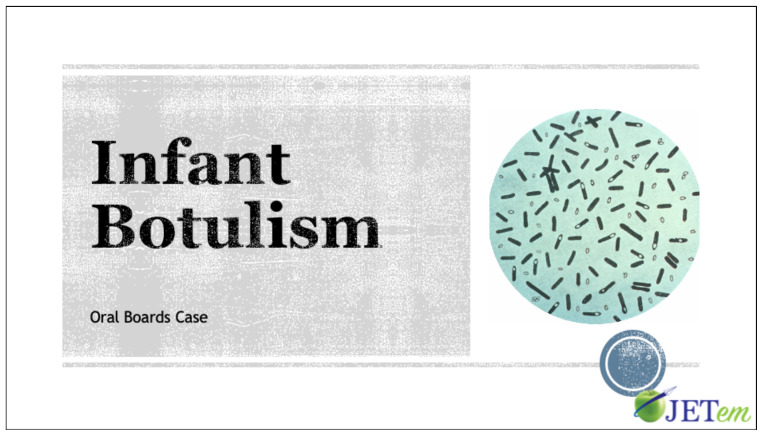
Please see associated PowerPoint file

## FOR EXAMINER ONLY

### Oral Case Summary

**Diagnosis: Infant Botulism**

**Case Summary:** The patient is a 6-month-old female (Lily Pond) who is brought from home by mother for lethargy. She had been doing well since birth but has been constipated and fatigued for the last two days. The patient and her siblings recently had a mild URI-like illness. The parents treated the patient with glycerin suppositories for the constipation and honey for her cough, respectively. The mother did notice that the child seemed to get tired easily when breastfeeding for the last two days and will only feed for 1–2 minutes at a time.

**Order of Case:** Patient arrives by private car with her mother. The learner performs the ABC’s. The learner recognizes that the patient is extremely lethargic, has poor muscle tone, and is bradypneic with an absent gag reflex. The learner asks for a point of care glucose. The learner asks to place the patient on the cardiac monitor, pulse oximonitor, and to establish an IV.

After performing the primary survey, the learner realizes that the infant needs to be intubated. The learner gives appropriate rapid sequence intubation medications, intubates, orders subsequent sedation, and asks for a portable chest x-ray (CXR). The learner may choose a size 4.0–4.5 ET tube with a size 1 blade (Mac, Miller, or video laryngoscopy. For rapid sequence induction (RSI) medications, the learner may choose etomidate (0.3 mg/kg), ketamine (1–2 mg/kg), or propofol (1mg/kg) and rocuronium (1 mg/kg), or an equivalent non-depolarizing neuromuscular blocking agent, while avoiding depolarizing agents such as succinylcholine. The learner can also verbalize how they would arrive at the correct equipment size and/or medication dosage using a Broselow tape or another equivalent calculator. Post-intubation sedation can include anxiolytics such as benzodiazepines and pain medication such as fentanyl or morphine with the dosing titrated per pharmacy to effect. The learner assesses the endotracheal (ET) tube position and only auscultates lung sounds on the right. The CXR demonstrates that the ET tube is in the right mainstem bronchus and the learner will need to pull back the tube 1–2 cm and order a repeat CXR. The repeat CXR will show adequate positioning of the ET tube at approximately 2 cm above the carina. If the learner pulls the ET back any more than 2 cm, the tube will become dislodged, requiring the learner to re-intubate and order a confirmatory CXR.

The learner will order and interpret additional tests. Pertinently, the electrocardiogram (EKG) will demonstrate sinus tachycardia at an approximate rate of 120 beats per minute, the complete blood count (CBC) will demonstrate a mild leukocytosis, the basic metabolic panel (BMP) will demonstrate mild acute kidney injury (AKI) consistent with dehydration, the venous blood gas (VBG) will demonstrate a mild respiratory acidosis, and the CT head will not demonstrate any acute intracranial pathology.

The learner recognizes that the history and physical exam are consistent with infant botulism and orders confirmatory direct botulinum toxin assay from the stool, serum, or gastric contents. Subsequently, the learner will be informed by a laboratory staff member that the testing samples will have to be sent to the state health department to be run. If the learner asks for the results, they will be prompted that “the results won’t be available for 3–5 days.” The learner does not delay the appropriate treatment and consults the IBTPP regarding the administration of botulinum antitoxin while waiting for results. If the learner orders BIG-IV but does not consult the IBTPP, then the pharmacist will contact the learner and notify them that this medication can only be obtained after speaking with the on-call physician at the IBTPP.

The learner explains everything to the mother.

**Disposition:** Admission to pediatric intensive care unit

**Critical Actions:**

1. Obtains point-of-care (POC) Glucose (data acquisition, systems-based practice)
2. Intubates the patient (problem-solving, patient care)
3. Recognizes botulism exposure and orders direct botulinum toxin assay from stool, serum, or gastric contents. (data acquisition, pathophysiology, systems-based practice)
4. Orders botulinum antitoxin (botulism immune globulin intravenous, ie. human BIG-IV) (problem-solving, patient care)
5. Admits to pediatric intensive care unit (patient care, systems-based practice)

### Historical Information

**Chief Complaint:** Lethargy

**History of present illness:** 6-month-old female (Lily Pond) presents with her mother for lethargy noted for the last two days. Mother reports that the patient has been more fatigued than usual which has been worsening over the last two days. The patient will no longer sit upright unassisted and has not been able to breastfeed for more than one to two minutes before becoming too tired to feed. Her last bowel movement was two days ago. The parents have been using glycerin suppositories without improvement. The patient has been making five wet diapers a day (usually makes nine daily). The parents have not noticed any fevers, chills, sweats, vomiting, diarrhea, fussiness/irritability (in fact, the mother will report the patient is barely crying at all), or rashes. All other review of systems are negative.

If specifically asked about recent sick contacts or recent illness, the mother will state that her other children and the patient had a mild cough and runny nose about two weeks ago which improved with honey. The mother will confirm that she treated the patient with honey in addition to her other children.

If asked specifically about medications, the mother will report that honey was used to treat a mild cough that the patient had about two weeks ago. The mother will also note that glycerin suppositories have been administered for the last two days for constipation.

**Past Medical History:** No chronic medical issues. Up to date on immunizations. Patient is breastfed every 3–4 hours.

**Birth History:** Born full term via normal spontaneous vaginal delivery (NSVD) and was discharged on hospital day 2 after birth.

**Past Surgical History:** None

**Patient’s Medications:** No regular medications. Glycerin suppositories as needed for constipation. Honey was given for a recent cough about 2 weeks ago.

**Allergies:** None

**Social history:** Lives with both parents and two siblings (4 and 6 years old). No exposure to secondhand smoke. Grandmother occasionally babysits.

**Family history:** Mother and Father are both healthy. Siblings do not have chronic medical issues.

### Physical Exam Information

[Table t2-jetem-8-3-o32] [Table t3-jetem-8-3-o32]

**Primary survey:**

- **Airway:** No gag reflex
- **Breathing:** Shallow breathing, respiratory rate of 12
- **Circulation:** Skin is dry and warm, cap refill < 2 seconds, equal pulses to bilateral femoral and brachial arteries.

**Physical examination:**

- **General appearance:** Well-developed, well-nourished, appears limp in mother’s arms
- **Head, eyes, ears, nose, throat (HEENT):**
  ○ **Head:** Atraumatic, normocephalic, fontanelle flat
  ○ **Eyes:** Dilated pupils, sluggish to react
  ○ **Ears:** Tymanic membranes (TMs) are clear (within normal limits)
  ○ **Nose:** Within normal limits
  ○ **Oropharynx/Throat:** Dry mucous membranes, pharynx clear, no cracked lips
- **Neck:** No lymphadenopathy, no thyromegaly
- **Chest:** Equal chest rise but shallow
- **Cardiovascular:** Normal heart sounds, regular rate and rhythm, peripheral pulses palpable and equal
- **Respiratory:** Equal to auscultation bilaterally but diminished in the bases, bradypneic with shallow respirations
- **Abdominal/GI:** Hypoactive bowel sounds, soft, no hepatomegaly, no masses
- **Genitourinary:** External genitalia normal, no diaper rash
- **Rectal:** Decreased tone, brown stool, guaiac negative
- **Extremities:** Floppy extremities, no deformities, no tenderness to palpation
- **Back:** No deformities, no tenderness to palpation
- **Neuro:** Awake and appears alert but sleepy, decreased muscle tone (unable to flex arms and legs against gravity when holding patient supine), 1+/4 deep tendon reflexes to bilateral upper and lower extremities, and does not withdraw from painful stimuli to any extremities (but does have weak cry to painful stimuli), weak suck reflex
- **Skin:** Warm, dry, no rashes or petechiae, normal skin turgor
- **Lymph:** Within normal limits

### Critical Actions and Cueing Guidelines

1. **Critical Action 1:** *Obtains POC Glucose*
  (Data acquisition, systems-based practice)
  This critical action is met by the learner recognizing that hypoglycemia is a differential diagnosis for a lethargic and weak infant and requesting a stat point of care glucose level.
  Cueing Guideline (if applicable): If the learner does not order a point of care glucose level prior to intubation, the mother should ask, “Do we really need to place a breathing tube? I think Lily just needs some nutrients through her IV.”
2. **Critical Action 2:** *Intubates the patient*
   (Problem-solving, patient care)
  This critical action is met by the learner recognizing airway/respiratory compromise and intubating the patient with appropriate rapid sequence intubation (RSI) medications, endotracheal (ET) tube size (4.0 to 4.5 – may say how they would determine the size if they do not know), and post-intubation sedation and analgesia (choose benzodiazepine and pain medication such as fentanyl or morphine). For RSI medications, the learner may choose etomidate (0.3 mg/kg), ketamine (1–2 mg/kg), or propofol (1mg/kg) and rocuronium (1 mg/kg) or an equivalent non-depolarizing neuromuscular blocking agent while avoiding depolarizing agents such as succinylcholine; the learner can also verbalize how they would arrive at the correct medication dosage. The learner should check the placement of the tube by asking for a color change on the colorimeter end-tidal carbon dioxide detector and check the pulse oximeter. The learner should listen over the bilateral lung fields and the epigastric area. The learner should troubleshoot the placement of the ET tube based on post-intubation auscultation and/or the first chest x-ray by pulling the tube back 1–2 cm. The learner should re-auscultate the lungs and order a repeat chest x-ray to confirm positioning. If the learner pulls the ET tube more than 2 cm, the ET tube will become dislodged and they will need to re-intubate the patient.
  Cueing Guideline (if applicable): If the learner fails to intubate the patient after the primary and secondary survey, the nurse should tell the learner, “The patient looks like she is struggling to breathe. What would you like to do?” Each strategy that the learner tries (such as: nasal cannula, high-flow oxygen, non-invasive mechanical ventilation, etc.) will not improve the patient’s bradypnea or work of breathing; however, the oxygenation will remain greater than 98%.
3. **Critical Action 3:** *Recognizes botulism exposure and orders direct botulinum toxin assay from stool, serum, or gastric contents*
   (Data acquisition, systems-based practice)
  This critical action is met by the learner’s ordering direct botulinum toxin assay after recognizing that the use of honey as an over-the-counter therapy in infants is a known risk factor for infant botulism. The laboratory staff may contact the learner to inform them that the test must be sent to the state health department in order to be performed and results will be unavailable for up to 5 days.
  Cueing Guideline (if applicable): If the learner does not order the direct botulinum toxin, the mother will ask, “What do you think caused Lily to get so sick?”
4. **Critical Action 4:** *Orders botulinum antitoxin (botulism immune globulin intravenous, ie, human BIG-IV)*
   (Problem-solving, patient care)
  This critical action is met by the learner’s recognizing infant botulism as the likely diagnosis and ordering botulinum antitoxin (botulism immune globulin intravenous, ie, human BIG-IV) before direct botulinum toxin assay results are available. If this is not first discussed with the IBTPP, then the pharmacist will notify the learner that this medication is only available after contacting and consulting the on-call IBTPP physician.
  Cueing Guideline (if applicable): If the learner does not order botulinum antitoxin, the nurse will tell the learner, “The lab tech called and said the test results for the botulinum toxin will not be ready for a few days.”
5. **Critical Action 5:** *Admits to pediatric intensive care unit (PICU)*
   (Patient care, system-based practice)
  This critical action is met by the learner’s giving a report to the pediatric intensivist.
  Cueing Guideline (if applicable): If the learner does not admit the patient, then 1) the nurse will remind the learner that all test results are back (except for the botulinum toxin assay will not be available for greater than 24 hours) and that a disposition needs to be made, OR 2) the mother will ask to be updated on the final plan and disposition for the patient.

## ORAL BOARDS ASSESSMENT

*Infant Botulism*

Learner: _________________________________________

**Critical Actions:**

□ Obtains POC Glucose (Data acquisition, systems-based practice)
□ Intubates the patient (Problem-solving, patient care)
□ Recognizes botulism exposure & orders direct botulinum toxin assay from stool, serum, or gastric contents (Data acquisition, systems-based practice)
□ Orders botulinum antitoxin (botulism immune globulin intravenous, ie, human BIG-IV) (Problem-solving, patient care)
□ Admits to pediatric intensive care unit (Patient care, system-based practice)

**Summative and formative comments:**

**Milestone assessment:** [Table t4-jetem-8-3-o32]

### Stimulus Inventory

[Table t5-jetem-8-3-o32]

#### Stimulus #1

##### Patient Information

[Table t6-jetem-8-3-o32]

#### Stimulus #2

##### Monitor Strip

[Fig f1-jetem-8-3-o32]

Image Source: Author’s own image.

#### Stimulus #3

##### 12 Lead EKG

[Fig f2-jetem-8-3-o32]

Image Source: Author’s own image.

#### Stimulus #4

##### Portable Chest X-ray

[Fig f3-jetem-8-3-o32]

Image Source: Qureshi P. Misplaced endotracheal tube. In: Radiopaedia. https://radiopaedia.org/cases/misplaced-endotracheal-tube-3?lang=us. CC BY-NC-SA 3.0.

#### Stimulus #5

##### Portable Chest X-ray

[Fig f4-jetem-8-3-o32]

Image Source: Herman L. Intubated pediatric chest xray. Own work. 2022. Used with permission.

#### Stimulus #6

##### Complete Blood Count (CBC)

[Table t7-jetem-8-3-o32]

#### Stimulus #7

##### Chemistry Basic Metabolic Panel (BMP)

[Table t8-jetem-8-3-o32]

#### Stimulus #8

##### Venous Blood Gas with Lactate

[Table t9-jetem-8-3-o32]

#### Stimulus #9

##### Urinalysis

[Table t10-jetem-8-3-o32]

#### Stimulus #10

##### CT Head without contrast

[Fig f5-jetem-8-3-o32]

Image Source: Herman L. Normal pediatric CT head. Own work. 2022. Used with permission.

#### Stimulus #11

##### Viral Swabs

[Table t11-jetem-8-3-o32]

## DEBRIEFING AND EVALUATION PEARLS

### Infant Botulism

[Fig f6-jetem-8-3-o32]
