# Supplementary material for: Infant Botulism
Source: J Educ Teach Emerg Med. 2023 Jul 31;8(3):O33–60. doi: 10.21980/J88350 (PMC10414984; doi:10.21980/J88350)
Supplement: Supplementary file 1 [file JETem-8-3-O32-supp1.pptx]

## Slide 1
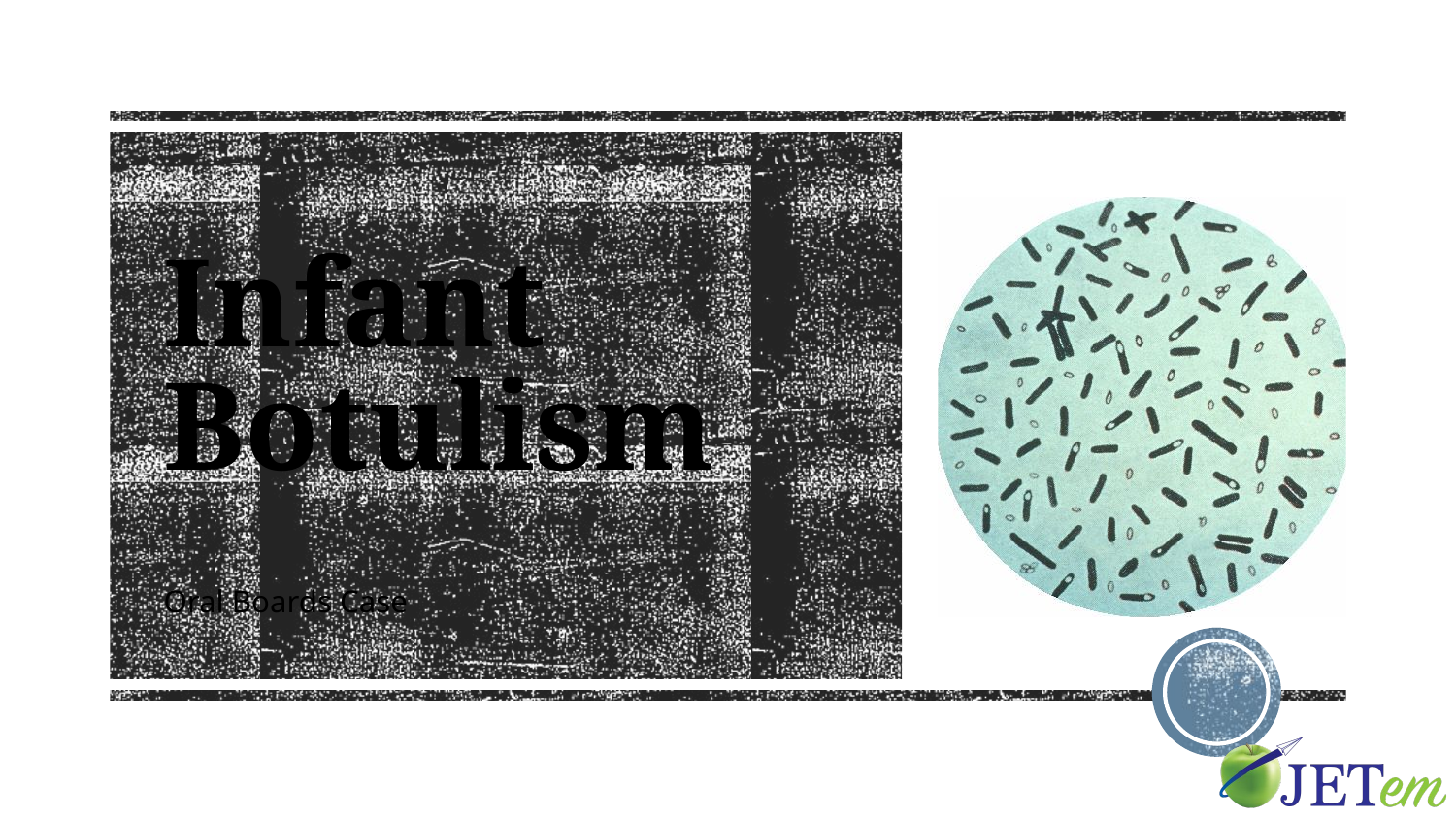

# Infant Botulism
Oral Boards Case

## Slide 2
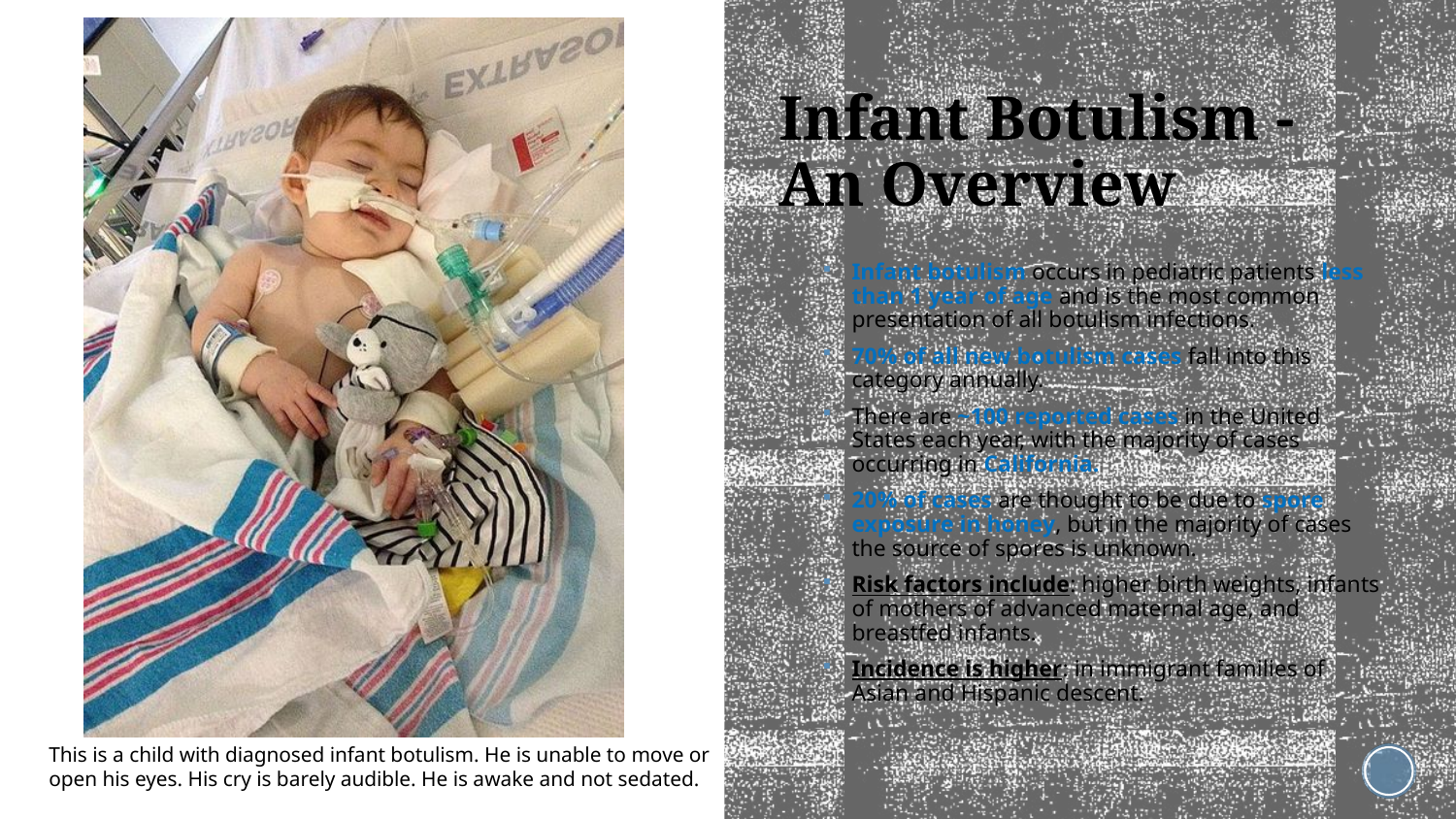

# Infant Botulism - An Overview
Infant botulism occurs in pediatric patients less than 1 year of age and is the most common presentation of all botulism infections.
70% of all new botulism cases fall into this category annually.
There are ~100 reported cases in the United States each year, with the majority of cases occurring in California.
20% of cases are thought to be due to spore exposure in honey, but in the majority of cases the source of spores is unknown.
Risk factors include: higher birth weights, infants of mothers of advanced maternal age, and breastfed infants.
Incidence is higher: in immigrant families of Asian and Hispanic descent.
This is a child with diagnosed infant botulism. He is unable to move or open his eyes. His cry is barely audible. He is awake and not sedated.

## Slide 3
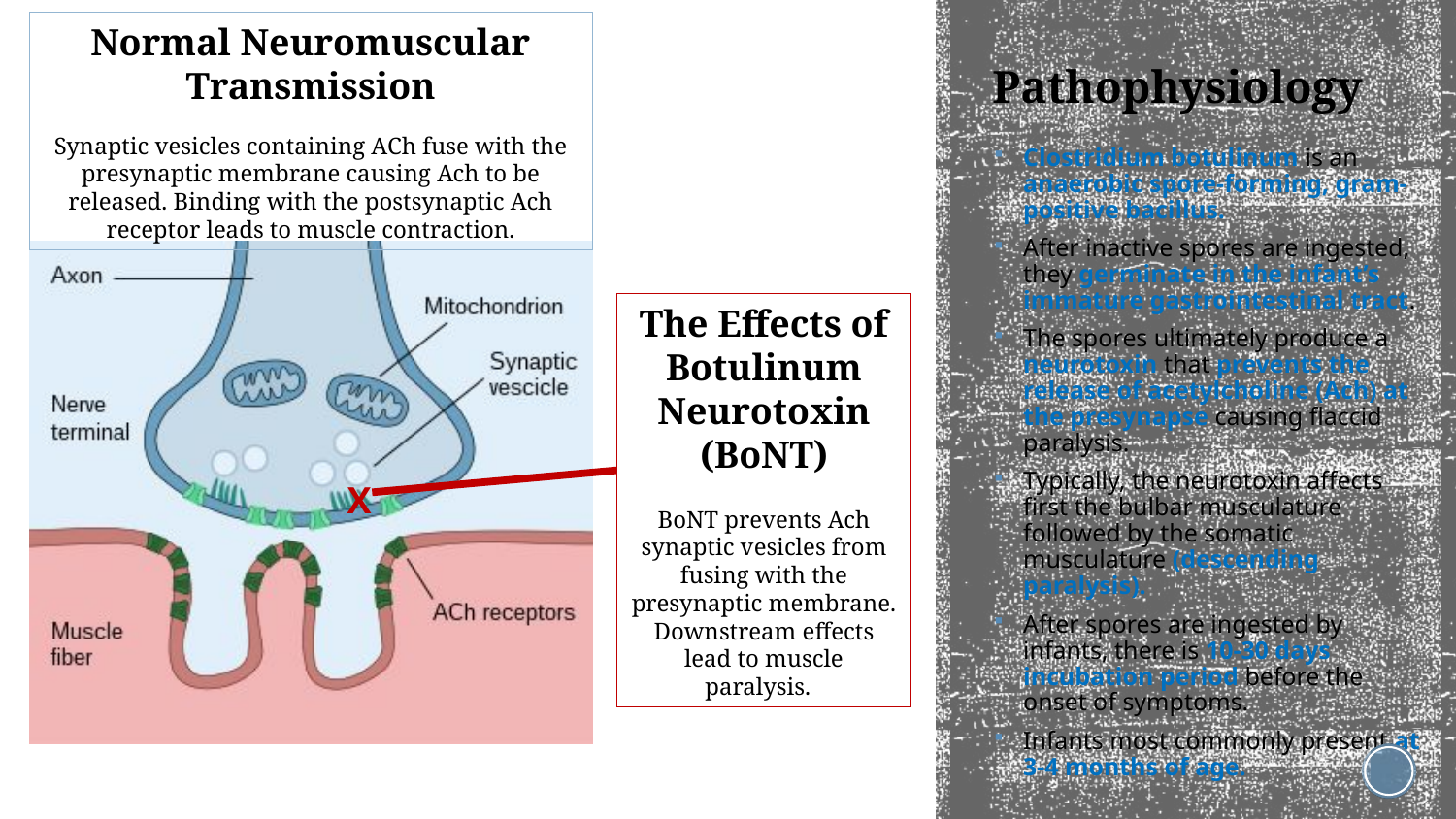

Normal Neuromuscular Transmission
Synaptic vesicles containing ACh fuse with the presynaptic membrane causing Ach to be released. Binding with the postsynaptic Ach receptor leads to muscle contraction.
# Pathophysiology
Clostridium botulinum is an anaerobic spore-forming, gram-positive bacillus.
After inactive spores are ingested, they germinate in the infant’s immature gastrointestinal tract.
The spores ultimately produce a neurotoxin that prevents the release of acetylcholine (Ach) at the presynapse causing flaccid paralysis.
Typically, the neurotoxin affects first the bulbar musculature followed by the somatic musculature (descending paralysis).
After spores are ingested by infants, there is 10-30 days incubation period before the onset of symptoms.
Infants most commonly present at 3-4 months of age.
The Effects of Botulinum Neurotoxin (BoNT)
BoNT prevents Ach synaptic vesicles from fusing with the presynaptic membrane. Downstream effects lead to muscle paralysis.
X

## Slide 4
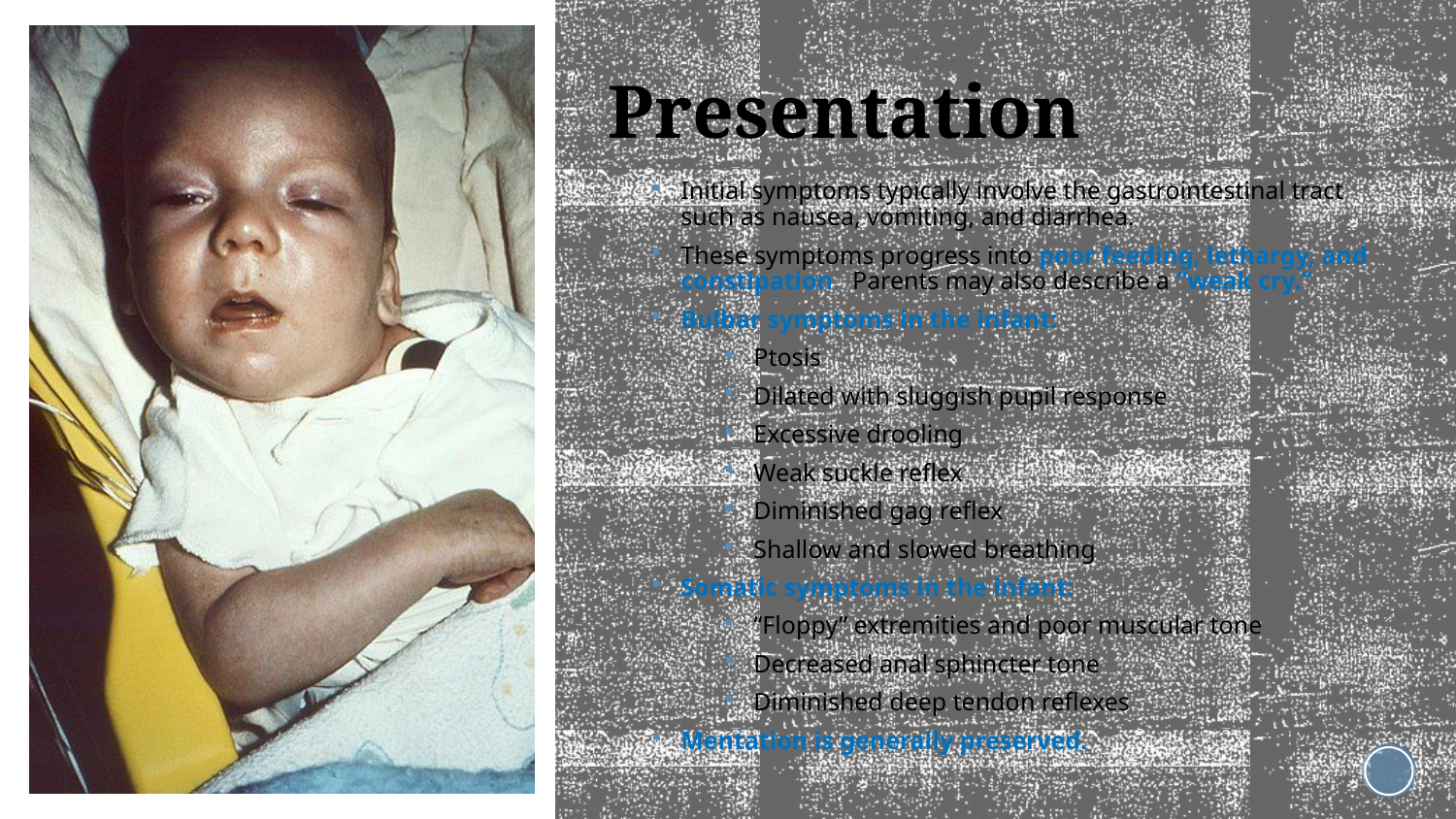

# Presentation
Initial symptoms typically involve the gastrointestinal tract such as nausea, vomiting, and diarrhea.
These symptoms progress into poor feeding, lethargy, and constipation. Parents may also describe a “weak cry.”
Bulbar symptoms in the infant:
Ptosis
Dilated with sluggish pupil response
Excessive drooling
Weak suckle reflex
Diminished gag reflex
Shallow and slowed breathing
Somatic symptoms in the infant:
“Floppy” extremities and poor muscular tone
Decreased anal sphincter tone
Diminished deep tendon reflexes
Mentation is generally preserved.

## Slide 5
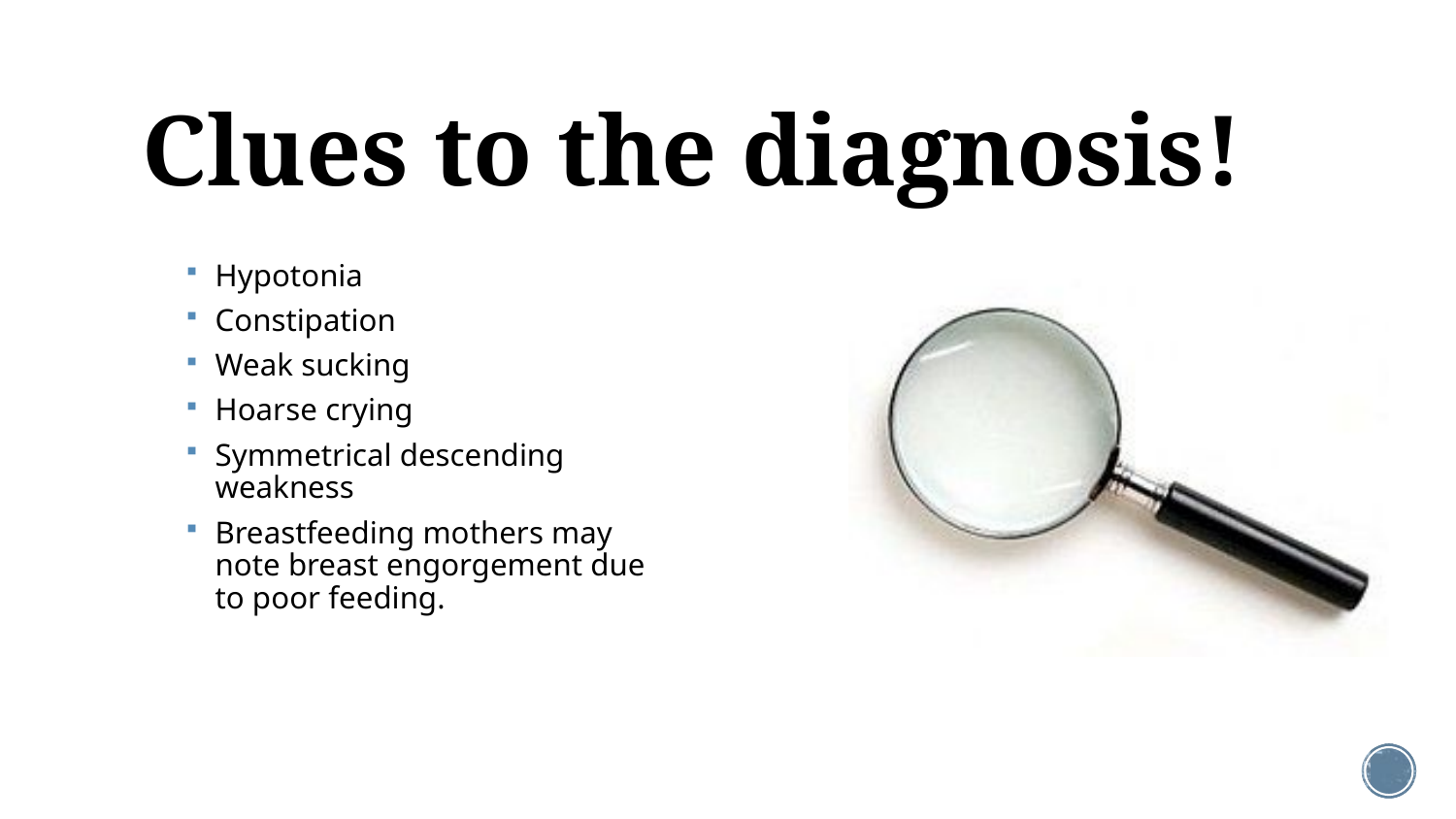

# Clues to the diagnosis!
Hypotonia
Constipation
Weak sucking
Hoarse crying
Symmetrical descending weakness
Breastfeeding mothers may note breast engorgement due to poor feeding.

## Slide 6
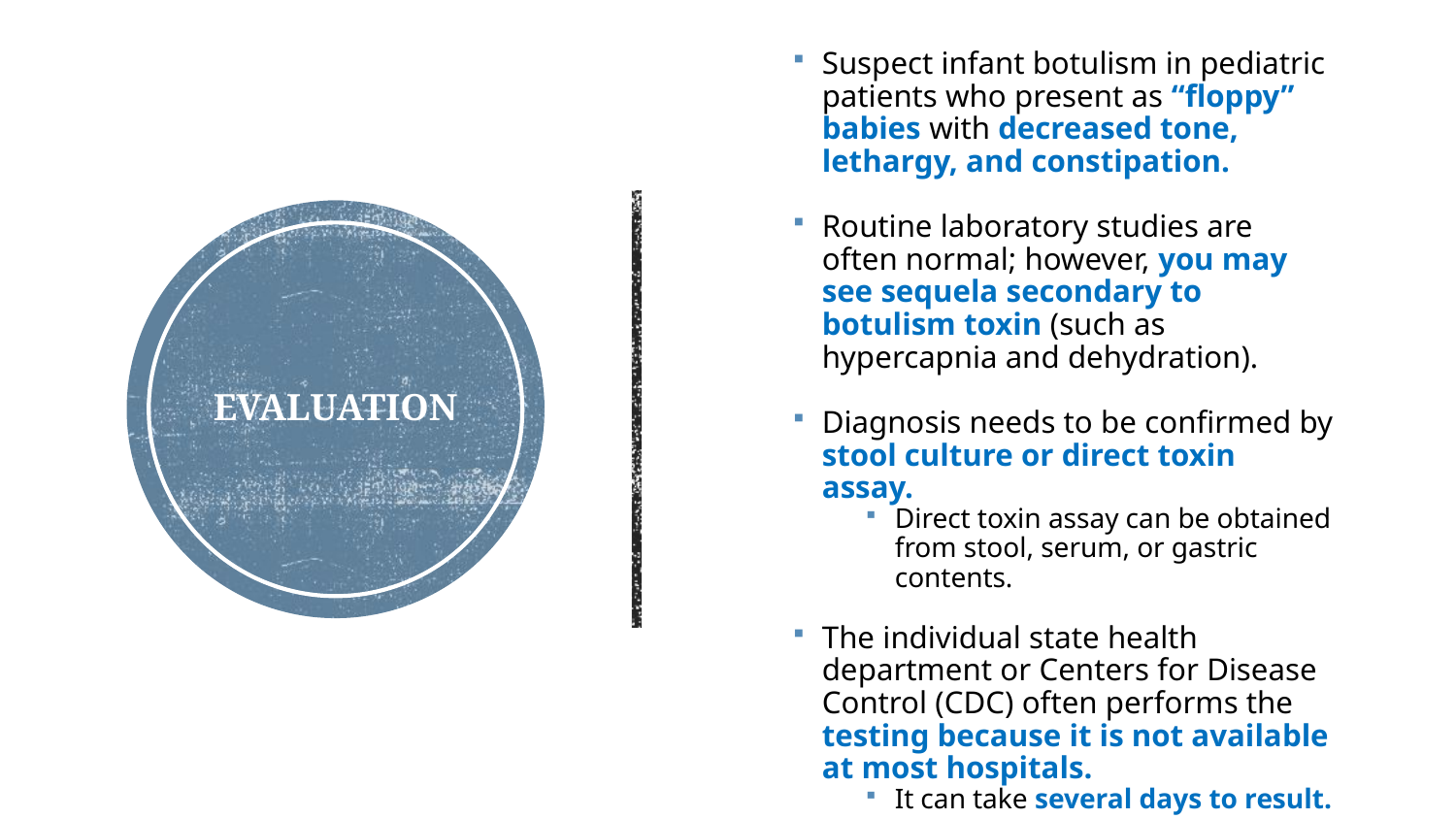

Suspect infant botulism in pediatric patients who present as “floppy” babies with decreased tone, lethargy, and constipation.
Routine laboratory studies are often normal; however, you may see sequela secondary to botulism toxin (such as hypercapnia and dehydration).
Diagnosis needs to be confirmed by stool culture or direct toxin assay.
Direct toxin assay can be obtained from stool, serum, or gastric contents.
The individual state health department or Centers for Disease Control (CDC) often performs the testing because it is not available at most hospitals.
It can take several days to result.
# Evaluation

## Slide 7
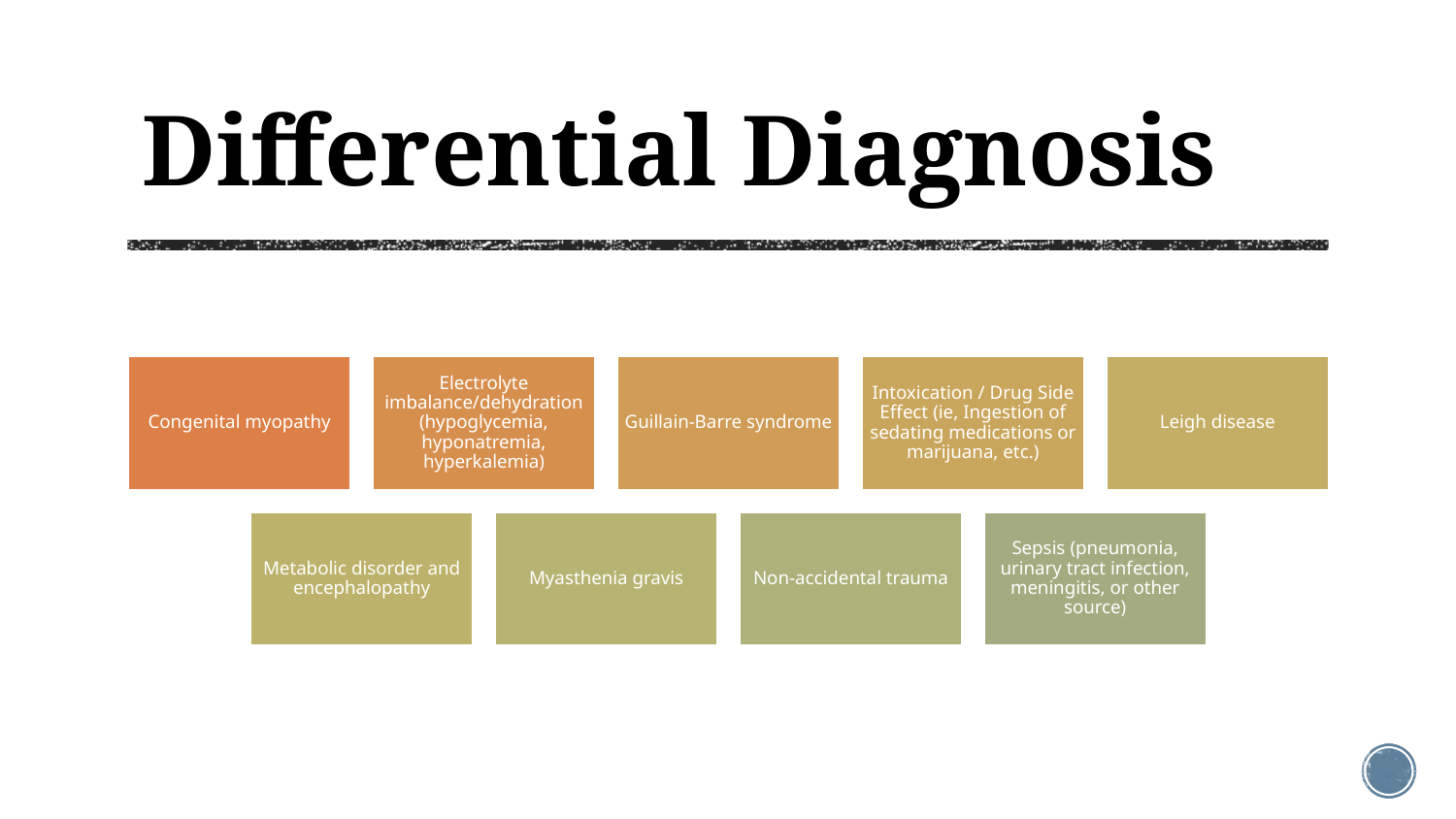

# Differential Diagnosis

## Slide 8
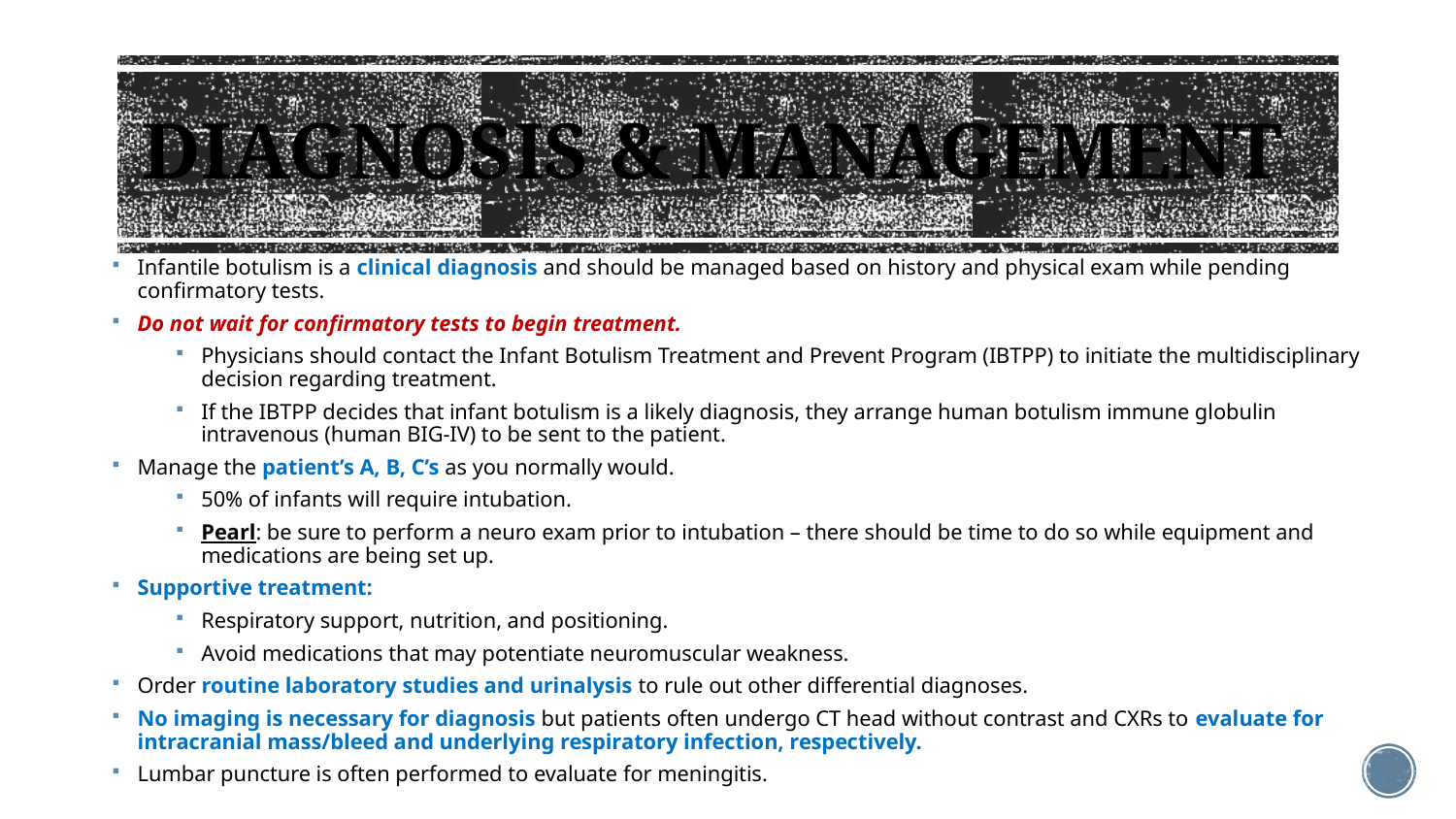

# Diagnosis & Management
Infantile botulism is a clinical diagnosis and should be managed based on history and physical exam while pending confirmatory tests.
Do not wait for confirmatory tests to begin treatment.
Physicians should contact the Infant Botulism Treatment and Prevent Program (IBTPP) to initiate the multidisciplinary decision regarding treatment.
If the IBTPP decides that infant botulism is a likely diagnosis, they arrange human botulism immune globulin intravenous (human BIG-IV) to be sent to the patient.
Manage the patient’s A, B, C’s as you normally would.
50% of infants will require intubation.
Pearl: be sure to perform a neuro exam prior to intubation – there should be time to do so while equipment and medications are being set up.
Supportive treatment:
Respiratory support, nutrition, and positioning.
Avoid medications that may potentiate neuromuscular weakness.
Order routine laboratory studies and urinalysis to rule out other differential diagnoses.
No imaging is necessary for diagnosis but patients often undergo CT head without contrast and CXRs to evaluate for intracranial mass/bleed and underlying respiratory infection, respectively.
Lumbar puncture is often performed to evaluate for meningitis.

## Slide 9
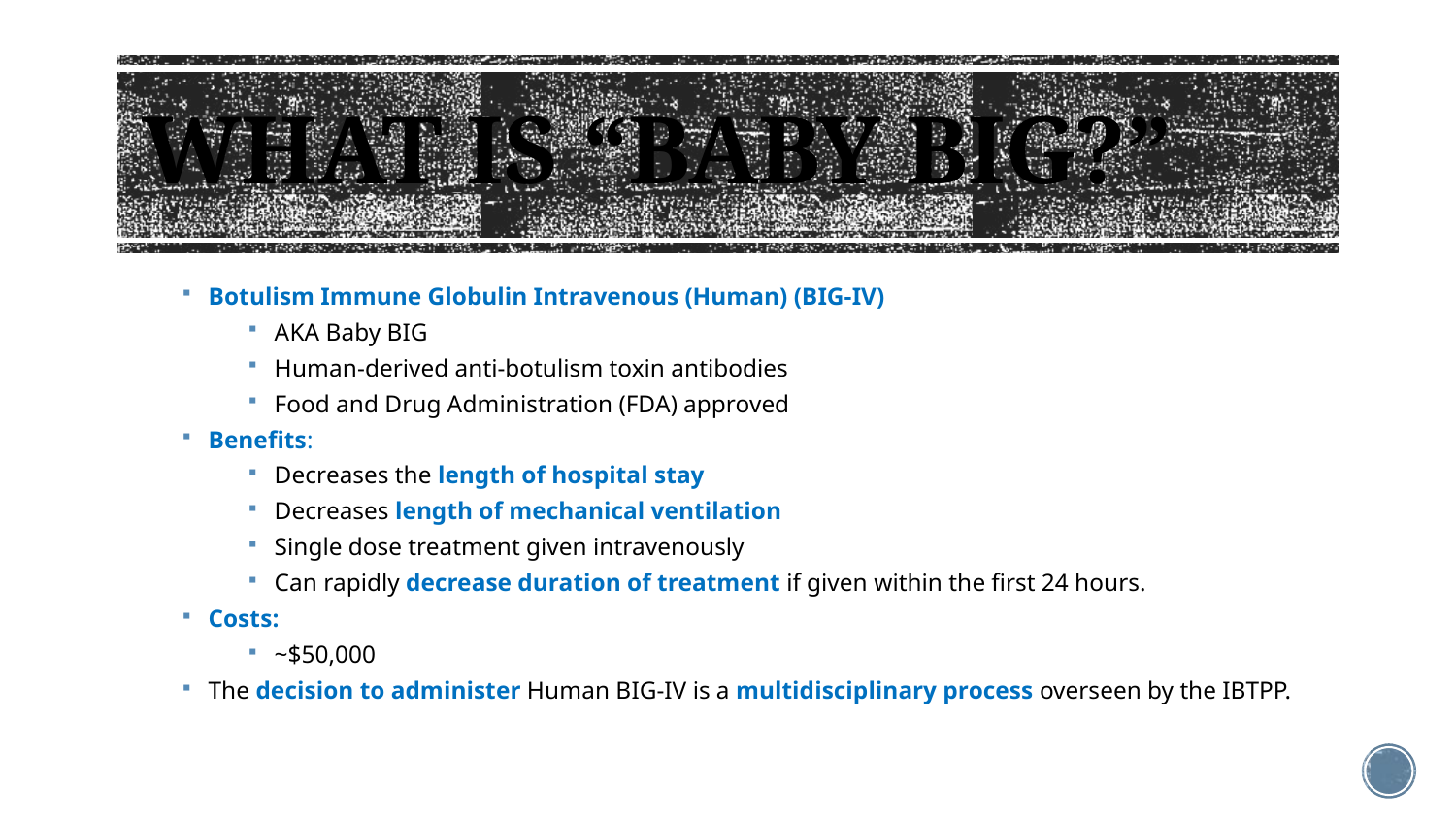

# What is “Baby BIG?”
Botulism Immune Globulin Intravenous (Human) (BIG-IV)
AKA Baby BIG
Human-derived anti-botulism toxin antibodies
Food and Drug Administration (FDA) approved
Benefits:
Decreases the length of hospital stay
Decreases length of mechanical ventilation
Single dose treatment given intravenously
Can rapidly decrease duration of treatment if given within the first 24 hours.
Costs:
~$50,000
The decision to administer Human BIG-IV is a multidisciplinary process overseen by the IBTPP.

## Slide 10
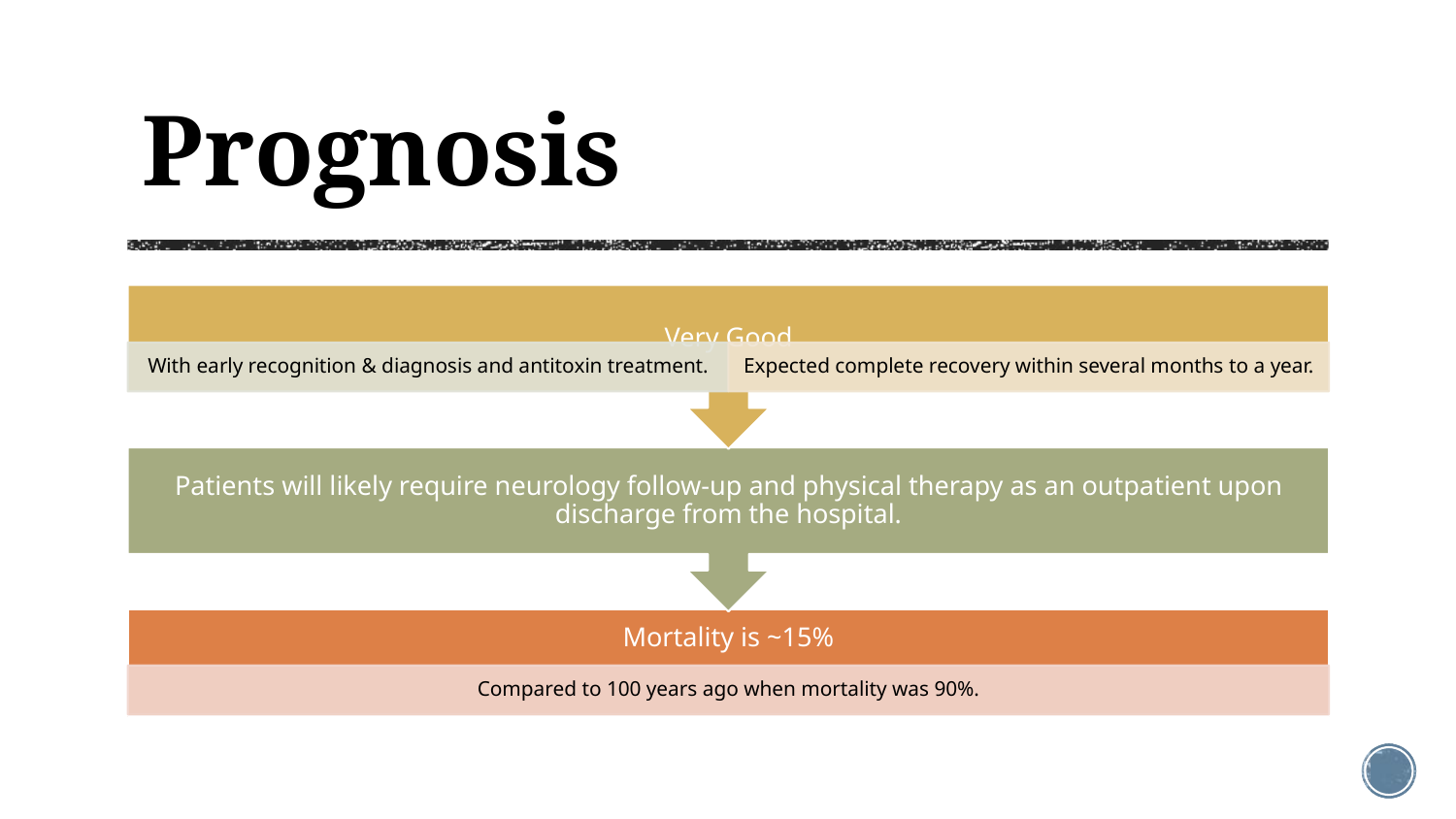

# Prognosis

## Slide 11
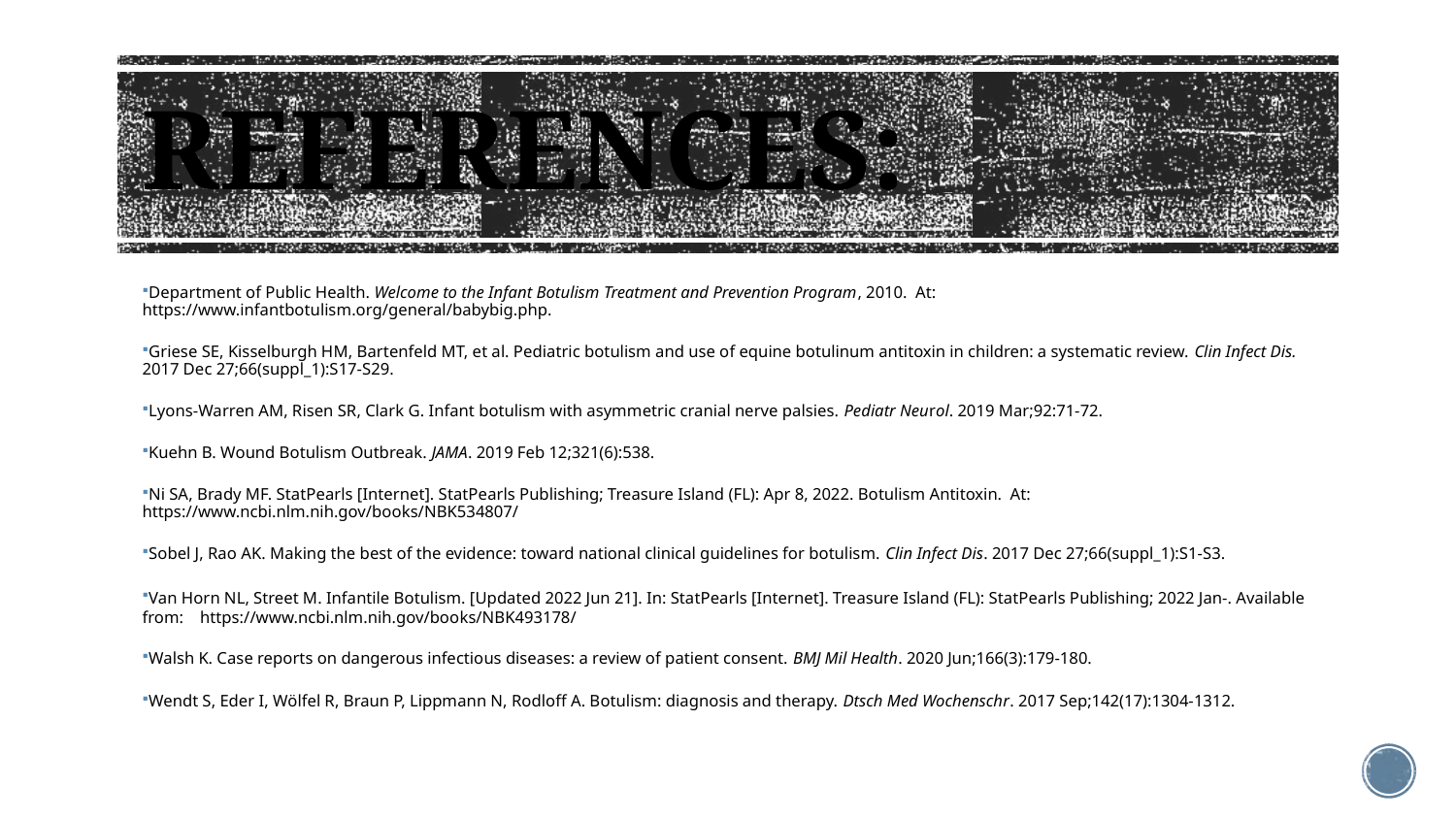

# References:
Department of Public Health. Welcome to the Infant Botulism Treatment and Prevention Program, 2010. At: https://www.infantbotulism.org/general/babybig.php.
Griese SE, Kisselburgh HM, Bartenfeld MT, et al. Pediatric botulism and use of equine botulinum antitoxin in children: a systematic review. Clin Infect Dis. 2017 Dec 27;66(suppl_1):S17-S29.
Lyons-Warren AM, Risen SR, Clark G. Infant botulism with asymmetric cranial nerve palsies. Pediatr Neurol. 2019 Mar;92:71-72.
Kuehn B. Wound Botulism Outbreak. JAMA. 2019 Feb 12;321(6):538.
Ni SA, Brady MF. StatPearls [Internet]. StatPearls Publishing; Treasure Island (FL): Apr 8, 2022. Botulism Antitoxin. At: https://www.ncbi.nlm.nih.gov/books/NBK534807/
Sobel J, Rao AK. Making the best of the evidence: toward national clinical guidelines for botulism. Clin Infect Dis. 2017 Dec 27;66(suppl_1):S1-S3.
Van Horn NL, Street M. Infantile Botulism. [Updated 2022 Jun 21]. In: StatPearls [Internet]. Treasure Island (FL): StatPearls Publishing; 2022 Jan-. Available from: https://www.ncbi.nlm.nih.gov/books/NBK493178/
Walsh K. Case reports on dangerous infectious diseases: a review of patient consent. BMJ Mil Health. 2020 Jun;166(3):179-180.
Wendt S, Eder I, Wölfel R, Braun P, Lippmann N, Rodloff A. Botulism: diagnosis and therapy. Dtsch Med Wochenschr. 2017 Sep;142(17):1304-1312.
